# Supplementary material for: FastqPuri: high-performance preprocessing of RNA-seq data
Source: BMC Bioinformatics. 2019 May 3;20:226. doi: 10.1186/s12859-019-2799-0 (PMC6500068; doi:10.1186/s12859-019-2799-0)
Supplement: Supplementary file 2 — Archive of FastqPuri. Archive containing all files needed to install and run FastqPuri v1.0.6. Date stamp March 22, 2019. (GZ 47,819 kb) [file 12859_2019_2799_MOESM2_ESM.gz › FastqPuri-1.0.6/html/globals_type.html]

FastqPuri: File Members


FastqPuri


- Ad\_seq
  : adapters.h
- Adapter
  : struct\_trimFilter.h
- Bfilter
  : bloom.h
- Bfkmer
  : bloom.h
- DS\_adap
  : trimDS.h
- Fa\_data
  : fa\_read.h
- Fa\_entry
  : fa\_read.h
- Fq\_read
  : fq\_read.h
- Info
  : stats\_info.h
- Iparam\_makeBloom
  : init\_makeBloom.h
- Iparam\_makeTree
  : init\_makeTree.h
- Iparam\_Qreport
  : init\_Qreport.h
- Iparam\_Sreport
  : init\_Sreport.h
- Iparam\_trimFilter
  : struct\_trimFilter.h
- Node
  : tree.h
- Split
  : str\_manip.h
- Stats\_TF
  : io\_trimFilter.h
- Stats\_TFDS
  : io\_trimFilterDS.h
- Tree
  : tree.h


---

Generated on Mon Mar 19 2018 23:42:01 for FastqPuri by  

 1.8.14
